# Cholesterol Perturbation in Mice Results in p53 Degradation and Axonal Pathology through p38 MAPK and Mdm2 Activation

**DOI:** 10.1371/journal.pone.0009999

**Published:** 2010-04-06

**Authors:** Qingyu Qin, Guanghong Liao, Michel Baudry, Xiaoning Bi

**Affiliations:** 1 Department of Basic Medical Sciences, College of Osteopathic Medicine of the Pacific (COMP), Western University of Health Sciences, Pomona, California, United States of America; 2 Neuroscience Program, University of Southern California, Los Angeles, California, United States of America; INSERM U862, France

## Abstract

Perturbation of lipid metabolism, especially of cholesterol homeostasis, can be catastrophic to mammalian brain, as it has the highest level of cholesterol in the body. This notion is best illustrated by the severe progressive neurodegeneration in Niemann-Pick Type C (NPC) disease, one of the lysosomal storage diseases, caused by mutations in the *NPC1* or *NPC2* gene. In this study, we found that growth cone collapse induced by genetic or pharmacological disruption of cholesterol egress from late endosomes/lysosomes was directly related to a decrease in axonal and growth cone levels of the phosphorylated form of the tumor suppressor factor p53. Cholesterol perturbation-induced growth cone collapse and decrease in phosphorylated p53 were reduced by inhibition of p38 mitogen-activated protein kinase (MAPK) and murine double minute (Mdm2) E3 ligase. Growth cone collapse induced by genetic (*npc1−/−*) or pharmacological modification of cholesterol metabolism was Rho kinase (ROCK)-dependent and associated with increased RhoA protein synthesis; both processes were significantly reduced by P38 MAPK or Mdm2 inhibition. Finally, *in vivo* ROCK inhibition significantly increased phosphorylated p53 levels and neurofilaments in axons, and axonal bundle size in *npc1−/−* mice. These results indicate that NPC-related and cholesterol perturbation-induced axonal pathology is associated with an abnormal signaling pathway consisting in p38 MAPK activation leading to Mdm2-mediated p53 degradation, followed by ROCK activation. These results also suggest new targets for pharmacological treatment of NPC disease and other diseases associated with disruption of cholesterol metabolism.

## Introduction

Axonal degeneration is a common feature of many neurodegenerative diseases, including Alzheimer's disease (AD), amyotrophic lateral sclerosis, Parkinson's disease, and Niemann-Pick type C (NPC) disease. NPC disease is caused by mutations in *NPC1* or *NPC2* gene, with late endosomal/lysosomal cholesterol accumulation as its characteristic pathologic feature. Intriguingly, although NPC proteins are ubiquitously distributed in the body and regulate intracellular cholesterol trafficking [1], the most prominent pathological feature of the disease is progressive neuronal death, particularly of neurons in cerebellum, cortex, thalamus and brainstem [reviewed in [2]]. Neuronal degeneration as well as other neuropathological features, including abnormal formation of meganeurites (spindle-shaped swelling in the initial segments of axons) and axonal spheroids, and inflammation have been reproduced in murine models of the disease [3], [4], [5], [6]. Interestingly, NPC pathology shares several features with AD pathology, including neurofibrillary tangles, autophagic/lysosomal dysfunction, inflammation, and cholesterol metabolism abnormalities [7], [8], [9], [10]. In some late onset NPC cases, amyloid plaques dependent on ApoE4 genotype are also present in certain parts of the brain [11]. Thus, NPC has often been used as a model system to study AD pathology.

Axonal degeneration together with intraneuronal cholesterol accumulation can be detected as early as postnatal day 9 in mice with mutant Npc1 proteins (*npc1−/−* mice) [12]. *In vitro* experiments with sympathetic neurons cultured from *npc1−/−* mice showed that, in parallel with cholesterol accumulation in late endosomes/lysosomes, cholesterol levels were decreased in the distal portions of axons [13]. Treatment of cultured hippocampal neurons from wild-type mice with the cholesterol transport inhibitor, U18666A, leads to a reduction in cholesterol content in axonal plasma membranes [14]. As inhibition of cholesterol synthesis induces axonal growth impairment [15], these results raise the possibility that cholesterol deficiency in axons may contribute to the axonal abnormalities found in NPC and other neurodegenerative diseases. In addition, defects in vesicle trafficking and abnormal autophagic/lysosomal function reported to be present in *npc1−/−* mice [7] could also affect axonal growth.

Axonal growth during development and axonal regeneration in adult nervous system depends on the motility of axonal growth cones. The dynamics as well as the directional motility of axonal growth cones are governed by both intrinsic factors and environmental clues. Guirland et al. recently showed that brain-derived neurotrophic factor (BDNF)-induced growth cone attraction was eliminated by membrane cholesterol depletion, and rescued by subsequent cholesterol restoration [16]. Likewise, growth cone repulsion induced by netrin-1 or semaphorin 3A was also disrupted by cholesterol depletion [16], indicating that membrane cholesterol is critically involved in the regulation of growth cone responses to environmental cues. We recently showed that the tumor suppressor protein p53 regulates growth cone motility through a transcription-independent mechanism [17]. In the present study we report that disruption of cholesterol egress from late endosomes/lysosomes induced by NPC1 deficiency or pharmacological manipulation resulted in growth cone collapse that was associated with abnormal activation of p38 mitogen-activated protein kinase (MAPK), which in turn led to Mdm2-dependent p53 degradation. Loss of p53 led to increased RhoA protein synthesis followed by Rho kinase activation and growth cone collapse. Our results indicate that this pathway plays a critical role in the pathogenesis of NPC and potentially other axonal diseases.

## Results

### Increased growth cone collapse and decreased levels of phosphorylated p53 in hippocampal neurons cultured from *npc1−/−* mice

**Figure 1:**
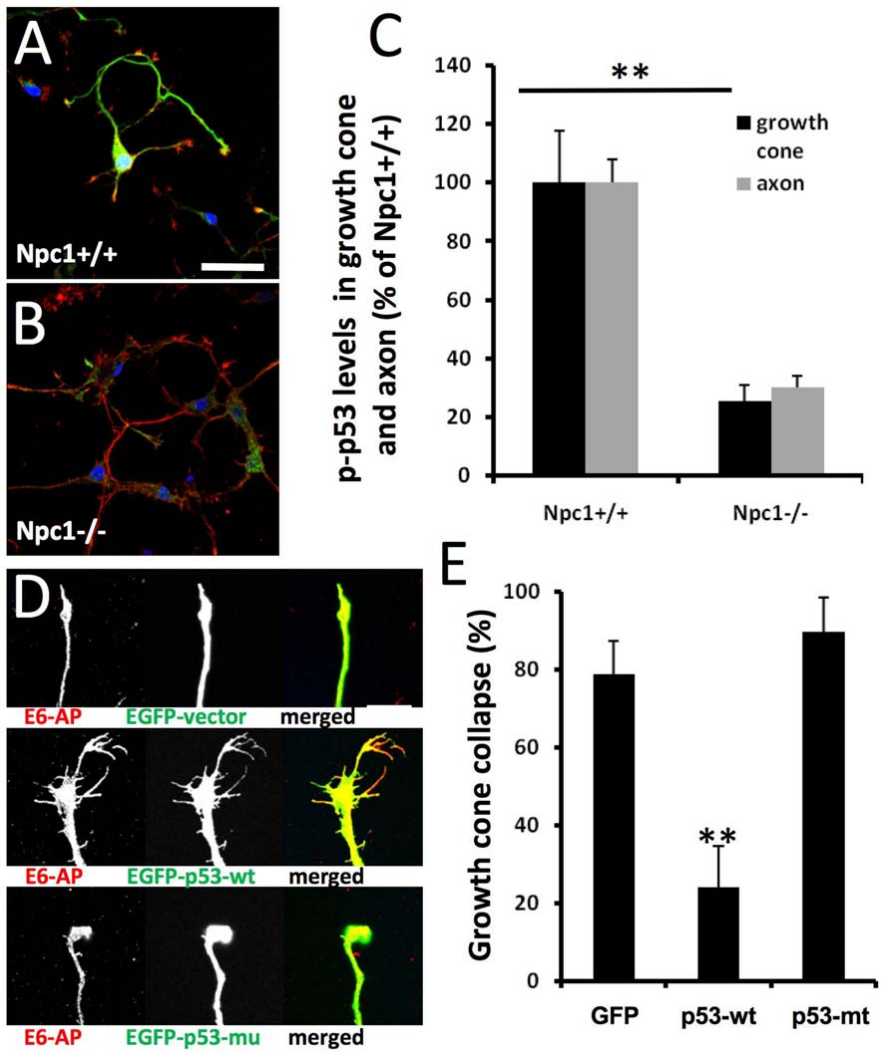
Deregulation of p53 is associated with abnormal axonal development in neurons with genetically- or pharmacologically-induced cholesterol transport perturbation. **A&B.** Immunofluorescence of p53 phosphorylated at Ser15 (p-p53, green) and E6-AP (red) in hippocampal neurons cultured from E18 *npc1+/+* (**A**) or *npc1−/−* embryos (**B**) and kept for 4 days *in vitro* (DIV4). Scale bar = 50 µm. **C.** Levels of p-p53 in axons and growth cones are decreased in DIV4 hippocampal neurons from *npc1−/−* mice. P-p53-immunoreactivity was quantified as described in Materials and Methods (n = 30 growth cones; **p<0.01 as compared to *npc1+/+* mice). **D&E.** Over-expression of wild-type p53 blocks growth cone collapse induced by cholesterol transport inhibition. **D.** Hippocampal neurons prepared from wild-type mice were transfected on DIV3 with either a EGFP vector, a EGFP-wild-type p53 (EGFP-p53-wt) vector, or a EGFP-p53 with R175H mutation (EGFP-p53-mu) vector, and treated with 1 µM U18666A for 18 h. Neurons were then fixed and processed for immunostaining with anti-E6AP (red). Scale bar = 20 µm. **E.** Quantitative analysis of U18666A-induced growth cone collapse (p<0.01 as compared to EGFP-vector transfected; n = 30 growth cones from 3 individual experiments).

Hippocampal neurons from E18 *npc1−/−* and *npc1+/+* embryos were cultured for 4 days *in vitro* (DIV) and processed for double-immunofluorescent staining with antibodies against E6-AP, an E3 ligase, and phosphorylated p53 (at Ser18 equivalent to human Ser15, hereafter referred to as p-p53); both proteins were highly expressed in axons and growth cones, as previously reported [17]. In cultured neurons from *npc1+/+* mice, high levels of p-p53 were observed mainly in cell bodies, axons and growth cones (Fig. 1A, green), whereas in those from *npc1−/−* mice, only low levels of p-p53 were found and mainly in cell bodies (Fig. 1B). Cultured hippocampal neurons from *npc1−/−* mice exhibited a much higher rate of growth cone collapse (78±2% vs 8±2%; n = 100 growth cones, p<0.01) with small growth cones and few or no filopodia, as compared to those from *npc1+/+* mice. Quantitative analysis indicated that levels of p-p53 immunoreactivity in axons and growth cones were decreased by about 80% as compared to wild-type values (Fig. 1C). Decreased axonal levels of p-p53 were also observed in brain tissues from 2-week old *npc1−/−* mice, especially in the striatum (Figure S1).

### U18666A-induced growth cone collapse was blocked by over-expression of wild-type p53

**Figure 2:**
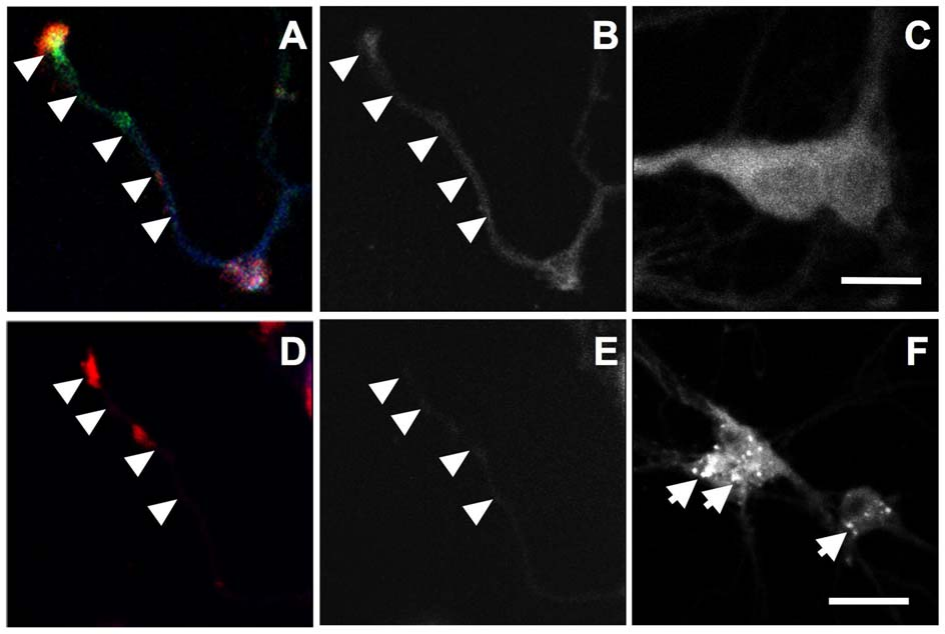
U18666A treatment decreases cholesterol levels in axons and growth cones. Cultured hippocampal neurons were treated with 1 µM U18666A (D-F) or DMSO (A–C) for 18 h before being processed for immunostaining with anti-E6-AP (red in A&D) and -p-p53 (green in A&D, to label axons and growth cones) antibodies followed by filipin staining (blue in A&D). Panels B and E show filipin staining in axons while C and F show staining in cell bodies. Scale bar = 20 µm.

As axonal growth cone collapse in hippocampal neurons from *npc1−/−* mice was associated with decreased levels of p-p53, we tested whether over-expression of wild-type p53 could reverse it. In this set of experiments, we used the amphiphilic amine U18666A to induce NPC-like phenotype in hippocampal neurons cultured from wild-type mice; U18666A has previously been used to induce NPC-like phenotype in various cultured cells, including neurons [18]. Treatment with 1 µM U18666A for 18 h induced growth cone collapse in about 80% of hippocampal neurons prepared from wild-type mice and transfected with an EGFP-vector or a vector containing p53 with the R175H mutation (a conformational mutation that is frequently found in tumor cells, resulting in lack of p53 function) (Fig. 1D&E). In neurons transfected with wild-type p53, the same treatment resulted in only 20% growth cone collapse (Fig. 1D&E). Wild-type p53 transfection also blocked growth cone collapse elicited by short-time (2 min) treatment with a higher concentration of U18666A (5 µM) (Figure S2). We previously showed that p53-R175H proteins formed aggregates in cell bodies and failed to be targeted to axons and growth cones in cultured hippocampal neurons [17], which may explain their lack of protective effects. To verify that treatment with 1 µM U18666A for 18 h disrupted cholesterol distribution, hippocampal neurons were stained with filipin, a fluorescent probe that has been widely used to stain cholesterol [19]. In vehicle-treated controls, filipin fluorescence was observed in cell bodies, axons (arrowheads), and growth cones (Fig. 2A–C). In U18666A-treated neurons, a marked decrease in fluorescence intensity was observed in axons and collapsed growth cones (Fig. 2D&E) in concurrence with the appearance of intensely labeled granules that resembled late endosomes/lysosomes in cell bodies (arrows in Fig. 2F).

### P38 MAPK and Mdm2 activation participated in U18666A treatment-induced p-p53 degradation and growth cone collapse

If decreased p-p53 levels were responsible for abnormal axonal development in neurons from *npc1−/−* mice, then what could lead to its down-regulation? In normal cells, p53 levels are tightly regulated by a negative feed-back loop between p53 and Mdm2: Mdm2 is a p53 target gene and Mdm2 activation results in p53 degradation. Since p38 MAPK has been shown to regulate both p53 [20] and Mdm2 [21], [22], we analyzed the roles of p38 MAPK and Mdm2 in regulation of p53 levels and growth cone morphology.

**Figure 3:**
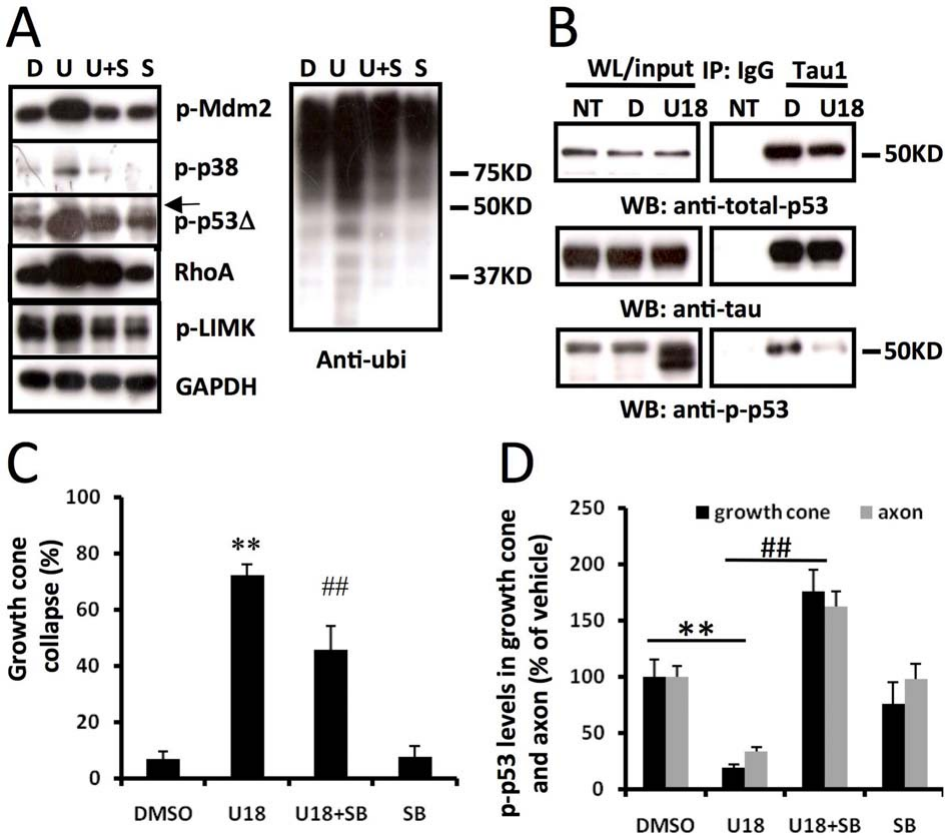
P38 MAPK activation is involved in growth cone collapse elicited by perturbation of cholesterol transport. **A.** Immunoblotting analysis of various proteins in cultured cortical neurons. Cortical neurons prepared from wild-type mice were treated on DIV4 with DMSO (D, vehicle), 5 µM U18666A (U), 5 µM U18666A plus p38 MAPK inhibitor, 1 µM SB203580 (U+S), or SB203580 alone (S). Shown are representative images of immunoblots probed with anti-phospho-Mdm2 (p-Mdm2), anti-phospho-p38 MAPK (p-p38), anti-phospho-p53 (p-p53, arrow), anti-RhoA, anti-phospho LIM Kinase (p-LIMK), anti-GAPDH (loading control), and anti-ubiquitin (Ubi) antibodies. U18666A treatment induced the appearance of a p-p53 immunopositive band (p-p53Δ) with a slightly smaller apparent molecular weight than native p-p53. **B.** Truncated p-p53 is not associated with axonal protein tau. Immunoprecipitation with Tau1 antibody or control IgG was performed as described in Material and Methods. Immunoprecipitated products and whole lysates (WL) were subjected to immunoblotting and blots were then probed with antibodies against total-p53, p-p53 or tau. U18666A U18) treatment resulted in a marked increase in p-p53Δ in whole lysates compared to DMSO treated or non-treated (NT). **C.** Inhibition of p38 MAPK reduced cholesterol perturbation-induced growth cone collapse. Quantification of growth cone collapse in DIV4 hippocampal neurons treated with DMSO or U18666A in the presence or absence of SB203580 pre-treatment was performed as described in Materials and Methods (**p<0.01 as compared to DMSO-treated, ##p<0.01 as compared to U18666A-treated; n = 100 growth cones from 3 individual experiments). **D.** Quantitative analysis of p-p53 levels in axons and growth cones of DIV4 hippocampal neurons. (**p<0.01 as compared to DMSO-treated and ##p<0.01 as compared to U18666A-treated; n = 25-40 growth cones from 3 individual experiments).

Immunoblotting analysis indicated that treatment of wild-type cortical neurons at DIV4 with 5 µM U18666A for 2 min induced a rapid decrease in p-p53 levels (arrow in Fig. 3A) with a corresponding increase in levels of a p-p53 immunopositive band with a slightly smaller molecular weight (thereafter referred to as p-p53 breakdown product, p-p53Δ) than in control samples. Since the p-p53Δ and p-p53 bands were very close in immunoblots and the former was the predominant band, we used p-p53Δ levels as an index of p-p53 degradation. We also used immunoprecipitation to determine whether p-p53 truncation affected its association with the microtubule-associated protein tau, a protein that is abundantly and exclusively expressed in axons. p53 labeled by either anti-p-p53 or anti-p53 antibodies was immunoprecipitated by Tau1 antibodies (Fig. 3B). Interestingly, although U18666A treatment resulted in a marked increase in p-p53Δ levels in whole lysates, p-p53Δ was absent in Tau1 pull-down products, suggesting that truncated p-p53 is not associated with axonal tau.

Immunoblotting results also showed that U18666A treatment markedly increased levels of Mdm2 phosphorylated at Ser166 (referred to as p-Mdm2 hereafter; Fig. 3A). Increased levels of p-p53Δ and p-Mdm2 were quantified using ImageJ program (Table 1). Levels of the dual-phosphorylated p38 MAPK (Thr180/Tyr182, hereafter referred to as p-p38), the active form of the enzyme [23], [24], were also increased in U18666A-treated neurons as compared to vehicle-treated (Fig. 3A), while levels of the non-phosphorylated p38 MAPK were not altered (Table 1). Immunofluorescent staining performed with antibodies against p-p38 and p-Mdm2 indicated that levels of these phosphoproteins were increased in axons and growth cones in U18666A-treated neurons, as compared to vehicle-treated controls (Figure S3). U18666A treatment also increased levels of RhoA and phosphorylated Lim kinase (p-LIMK) (Fig. 3A).

**Table 1:** Effects of p38 MAPK and Mdm2 inhibitors on U18666A-induced changes in various proteins.

	DMSO	U18666A	U18666A + SB203580	SB203580	U18666A + Mdm2_In	Mdm2-In
**p-Mdm2**	100	549±21**	100±2^##^	92±1	476±8	100±4
**p-p38**	100	493±24**	99±3^##^	96±24	472±19	108±12
**t-p38**	100	107±1	98±3	99±5	107±1	104±0
**p-p53Δ**	100	775±29**	97±6^##^	94±6	280±23^##^	106±2
**t-p53**	100	94±1	103±1	107±2	98±4	104±1
**Ubiquitin**	100	435±14 **	93±6^##^	95±7	117±5^##^	102±5
**RhoA**	100	418±16**	216±21^##^	96±0	114±6^##^	105±1
**p-LIMK**	100	411±15**	101±3^##^	100±3	147±10^##^	117±6
**p-4EBP1**	100	389±7**	276±19^#^	99±3	327±3^##^	104±2

**p<0.01 as compared to DMSO-treated;

#p<0.05 and ##p<0.01 as compared to U18666A-treated;

n = 3–6 from 3 individual experiments.

**Figure 4:**
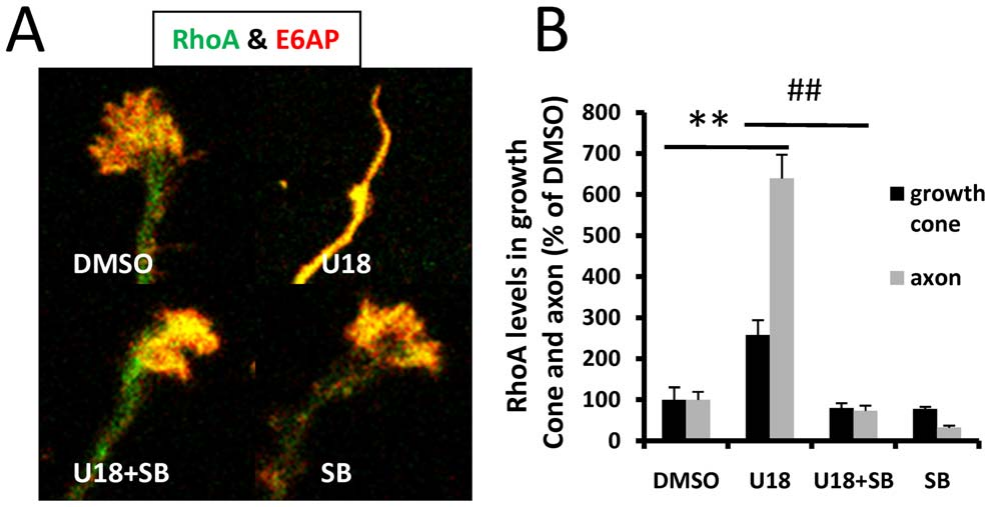
Inhibition of p38 MAPK blocked U18666A-induced increase in RhoA expression in axons and growth cones. Wild-type hippocampal neurons were treated at DIV 4 with DMSO or U18666A in the presence or absence of SB203580 (SB) pre-treatment and processed for immunostaining with anti-RhoA (green) and anti-E6-AP (red) antibodies as described in Materials and Methods. **A.** Representative images. **B.** Quantitative analysis of RhoA levels in axons and growth cones (**p<0.01 as compared to DMSO-treated and ##p<0.01 as compared to U18666A-treated; n = 25-40 growth cones from 3 individual experiments).

**Figure 5:**
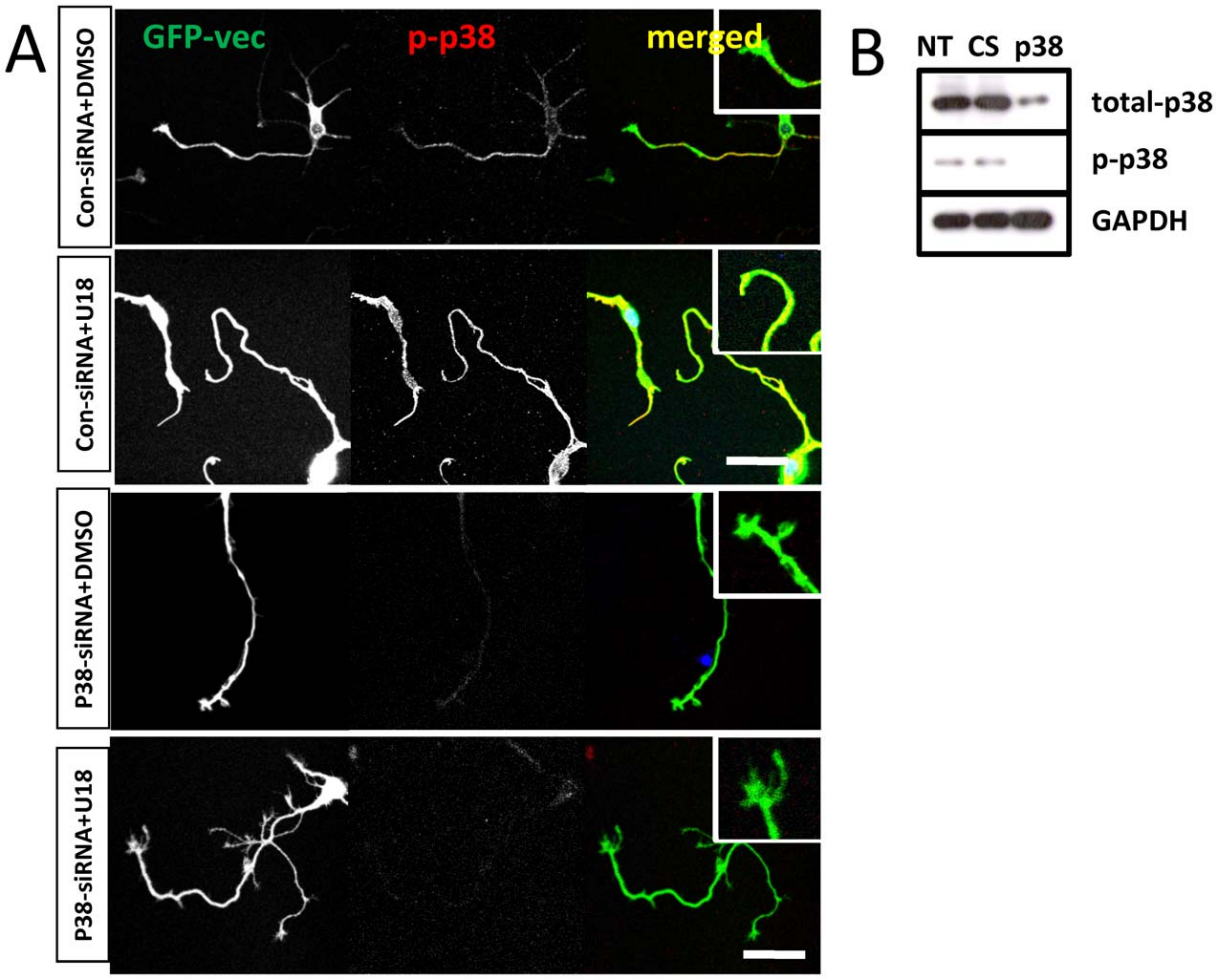
P38 MAPK specific siRNAs reduce U18666A-induced growth cone collapse. **A.** Hippocampal neurons cultured from wild-type mice were transfected with a set of siRNAs specific for p38 MAPK or control siRNAs and with a GFP vector on DIV3 and treated with U18666A on DIV 4 before being fixed and processed for immunostaining with anti-phospho-p38 (p-p38, red) antibodies. Inserts show enlarged images of growth cones. Application of p38 MAPK siRNAs, but not control siRNA, markedly reduced p-p38 immunoreactivity and U18666A-induced growth cone collapse. Results are representative of 3-4 culture dishes from 2 independent experiments. Scale bar = 50 µm. B. Immunoblotting analysis of p38 knock-down by siRNA. Cortical neurons transfected with p38 MAPK specific or control siRNAs (CS) on DIV3 were collected on DIV4 and processed for immunoblotting with anti-total p38, -p-p38, or GAPDH (loading control). Treatment with p38 MAPK specific siRNAs, but not control siRNA reduced both total p38 and p-p38 by 90% as compared to non-treated (NT).

To determine whether p38 MAPK activation was critically involved in U18666A-induced growth cone collapse, we tested the effects of a widely used p38 MAPK inhibitor, SB203580. Pre-incubation of cultured neurons with 1 µM SB203580 for 2 h before treatment with U18666A partially, but significantly, reduced growth cone collapse elicited by U18666A (Fig. 3C). P38 MAPK inhibition also markedly reduced Mdm2 and p38 MAPK phosphorylation, p-p53 degradation, and RhoA increase resulting from U18666A treatment. The blocking effects of SB203580 on p-p53 truncation (Fig. 3D) and RhoA increase (Fig. 4A&B) were even more evident in axons and growth cones when analyzed with immunohistochemistry. Immunoblots probed with anti-ubiquitin (Ubi) and anti-p-LIMK antibodies indicated that p38 MAPK inhibition also reduced U18666A treatment-induced increases in protein ubiquitination and LIMK phosphorylation (Fig. 3A; Table 1). Finally, U18666A-induced growth cone collapse was blocked by a set of siRNAs specific for p38 MAPK but not by control siRNAs, which further confirmed the involvement of this kinase in this process (Fig. 5). P38 MAPK siRNAs alone did not significantly modify growth cone morphology (Fig. 5A). The reduction of p-p38 levels by siRNA treatment was also confirmed by immunoblotting (Fig. 5B).

**Figure 6:**
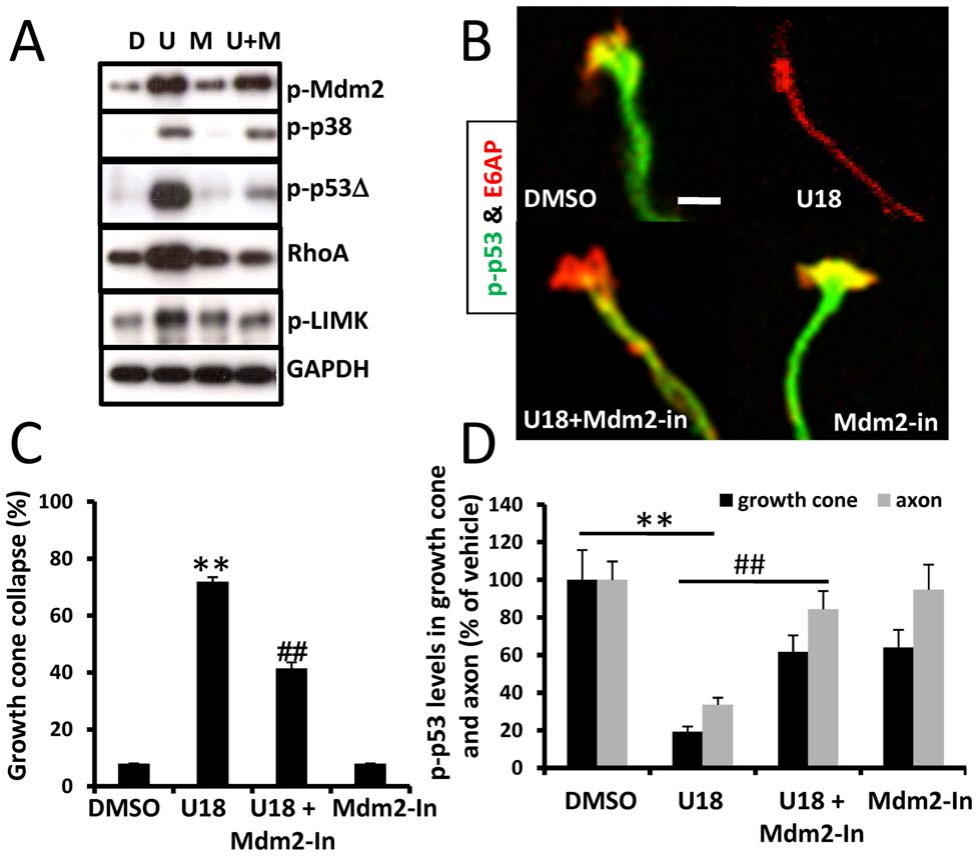
Mdm2 activation is involved in U18666A-induced p53 degradation and growth cone collapse. **A.** Mdm2 inhibition blocked U18666A treatment-induced p-p53 truncation and ROCK activation. Cultured cortical neurons were treated with DMSO (D) or U18666A (U) in the presence or absence of pre-treatment with an Mdm2 inhibitor (M). Shown are representative images of immunoblots probed with anti-phospho-Mdm2 (p-Mdm2), anti-phospho-p53 (p-p53), anti-phospho-p38 MAPK (p-p38), anti-RhoA, anti-phospho LIM Kinase (p-LIMK), and anti-GAPDH (loading control) antibodies. Mdm2 inhibitor (Mdm2-In) blocked U18666A-induced increases in p-p53Δ, RhoA, and p-LIMK, but not in pMdm2 or p-p38. **B&C.** Mdm2 inhibition blocked U18666A treatment-induced growth cone collapse. DIV4 hippocampal neurons treated with DMSO or U18666A (U18) in the presence or absence of Mdm2 inhibitor pre-treatment (Mdm2-In) were processed for immunostaining with anti-p-p53 (green) and -E6AP (red) antibodies. **B.** Representative images. Scale bar = 20 µm. **C.** Quantitative analysis of growth cone collapse. (**p<0.01 as compared to DMSO treated, ##p<0.01 as compared to U18666A treated; n = 100 growth cones from 3 individual experiments). **D.** Quantitative analysis of p-p53 levels in axons and growth cones of DIV4 hippocampal neurons (**p<0.01 as compared to DMSO-treated and ##p<0.01 as compared to U18666A-treated; n = 25-40 growth cones from 3 individual experiments).

The critical role of Mdm2 in U18666A-induced growth cone collapse was further tested with an Mdm2 specific inhibitor (Mdm2-in); pretreatment with 1 µM Mdm2-in significantly reduced U18666A-induced growth cone collapse (Fig. 6B&C; p<0.01, n = 100 growth cones). Immunoblotting results showed that the Mdm2 inhibitor also significantly reduced the increase in p-p53Δ (Fig. 6A; Table 1). Image analysis indicated that the Mdm2 inhibitor significantly reduced U18666A-induced decrease in p-p53 levels in axons and growth cones (Fig. 6D; p<0.01, n = 25–40 neurons). Mdm2 inhibition also blocked U18666A-induced increase in RhoA levels (Fig. 6A). Immunohistochemical analysis showed that in U18666A plus Mdm2 inhibitor-treated neurons, RhoA levels (expressed as % of vehicle treated) in axons and growth cones were reduced from 640±56% to 67±16% and 257±37% to 138±24% (mean ± SEM; p<0.01 when compared to U18666A-treated; n = 25–40 from 3 individual experiments), respectively. Mdm2 inhibitor alone did not significantly change RhoA expression in either axons (102±15%) or growth cones (136±20%). Mdm2 inhibition did not alter U18666A-induced phosphorylation of either Mdm2 or p38 (Fig. 6A).

### ROCK inhibition reduced U18666A-induced growth cone collapse and p-p53 truncation

**Figure 7:**
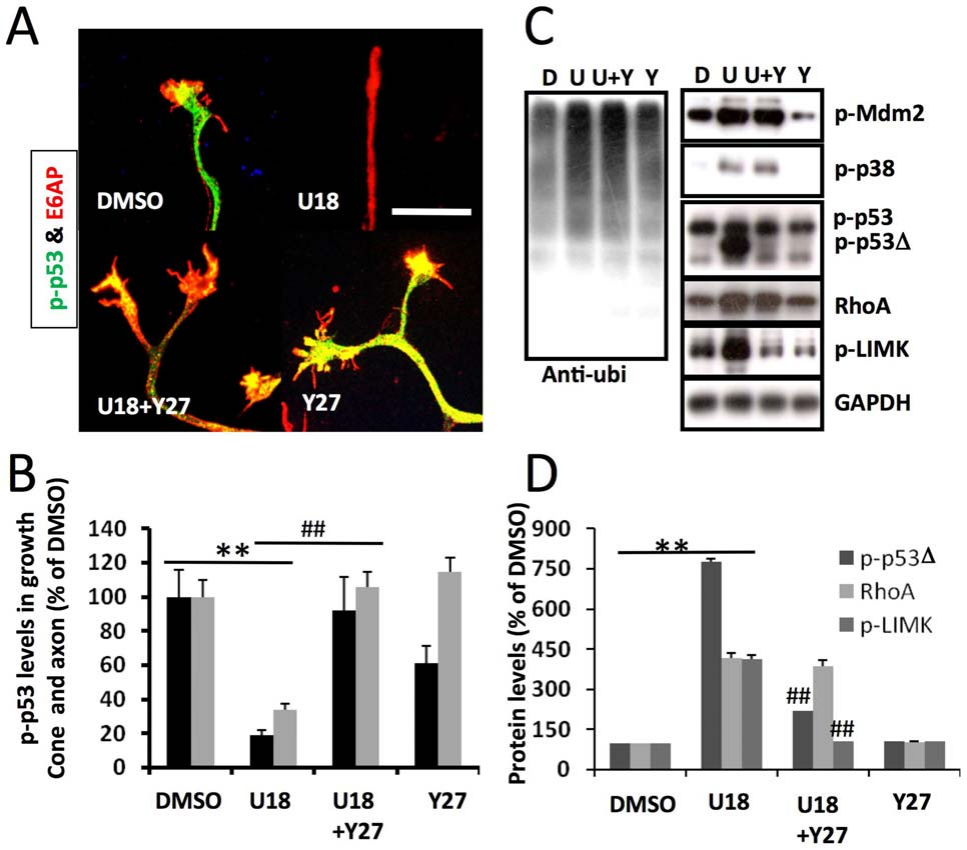
ROCK inhibition blocks U18666A-induced p-p53 decrease and rescues growth cones in hippocampal neurons cultured from wild-type mice. **A.** Immunofluorescence analysis of p-p53 (green) and E6-AP (red) distribution and growth cone morphology in cultured wild-type hippocampal neurons treated with DMSO or U18666A (U18) in the absence or presence of 10 µM Y27632 (Y27). Scale bar = 20 µm. **B.** Quantitative analysis of p-p53 levels in axons and growth cones of DIV4 hippocampal neurons (**p<0.01 as compared to DMSO-treated and ##p<0.01 as compared to U18666A-treated; n = 25-40 growth cones from 3 independent experiments). **C&D.** Immunoblotting analysis of various proteins in cultured cortical neurons treated with DMSO (D) or U18666A (U) in the presence or absence of Y27632 (Y). **C.** Representative images of immunoblots probed with antibodies against ubiquitin (Ubi), phospho-Mdm2 (p-Mdm2), phospho-p38 MAPK (p-p38), phospho-p53 (p-p53), RhoA, phospho LIM Kinase (p-LIMK), and GAPDH (loading control). **D.** Quantitative analysis of p-p53Δ, RhoA, and p-LIMK (**p<0.01 as compared to DMSO-treated; ##p<0.01 as compared to U18666A-treated; n = 3–6 from 3 individual experiments).

Rho kinase is critically involved in growth cone collapse and we previously showed that growth cone collapse induced by inhibition of p53 with pifithrin-μ was rescued by ROCK inhibitors [17]. Immunoblotting and immunohistochemical results showed that U18666A treatment induced a marked increase in RhoA levels, which was blocked by inhibition of p38 MAPK and Mdm2. To further test the role of the Rho-ROCK signaling pathway in U18666A-induced growth cone collapse, we pre-treated cultured neurons with the widely used specific ROCK inhibitor, Y27632 that has been shown to inhibit the ROCK family 100 times more potently than other kinases, including protein kinase C, cAMP-dependent kinase, and myosin light chain kinase [25]. Pre-incubation of wild-type hippocampal neurons at DIV4 with Y27632 (10 µM) for 2 h before treatment with 5 µM U18666A for 2 min significantly reduced U18666A-induced growth cone collapse (Fig. 7A; 41±1% vs. 72±2%; p<0.01, n = 100 growth cones). Y27632 pretreatment also reversed U18666A-induced decrease in p-p53 immunoreactivity in axons and growth cones (Fig. 7A&B). Similar results were obtained following pre-treatment with 1 µM Y27632 (not shown). The involvement of ROCK was further tested by using another inhibitor, H1152; pre-treatment with 100 nM H1152 for 3 h also blocked U18666A-induced growth cone collapse and decrease in p-p53 immunoreactivity (Figure S4). Immunoblotting results indicated that pre-treatment with Y27632 did not block U18666A-induced increase in p38 and Mdm2 phosphorylation, protein ubiquitination and RhoA levels (Fig. 7C&D), but significantly reduced U18666A-induced increase in levels of phosphorylated LIM kinase, an enzyme downstream of ROCK (p-LIMK; Fig. 7C). These results suggest that ROCK activation is downstream from p38 and Mdm2 activation and RhoA up-regulation.

Intriguingly, ROCK inhibition also significantly reduced p-p53 truncation. We previously showed that growth cone collapse and decreased levels of p-p53 in axons and growth cones induced by p53 inhibition were also blocked by Y27632. Together, these results suggest a mutual inhibitory regulation between p53 and ROCK. We had also previously shown that p53 in axons and growth cones was recognized by a “mutant” conformation specific antibody but not by the “wild-type” conformation specific antibody [17]. Treatment with U18666A markedly reduced levels of “mutant” p53 in axons and growth cones, an effect also blocked by Y27632 (Figure S5).

### ROCK inhibition reduced axonal abnormality of *npc1−/−* mice *in vitro* and *in vivo*

**Figure 8:**
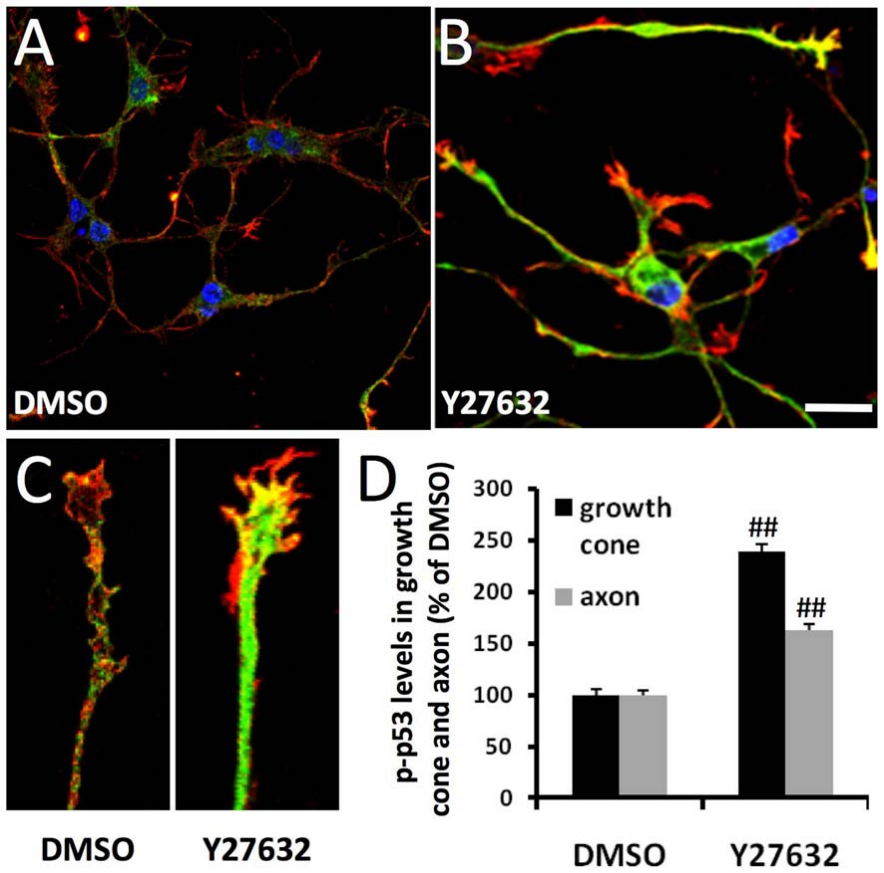
ROCK inhibition increases p-p53 levels and rescues growth cones in cultured hippocampal neurons from *npc1−/−* mice. **A–C.** Immunofluorescence of p-p53 (green) and E6-AP (red) in cultured *npc1−/−* hippocampal neurons treated with DMSO (**A**) or Y27632 (**B**). Scale bar = 50 µm. High power images of growth cones are shown in **C.** Hippocampal neurons were prepared from E18 *npc1−/−* embryos and treated with 0.01% DMSO or 10 µM Y27632 (ROCK inhibitor) on DIV3 for 24 h before being processed for immunofluorescence staining. **D.** Quantitative analysis of p-p53 levels in axons and growth cones (n = 30 growth cones; ##p<0.01 as compared to values in DMSO-treated neurons from *npc1−/−* mice).

We next tested whether ROCK activation was involved in spontaneous growth cone collapse in neurons with genetic Npc1 deficiency. Hippocampal neurons cultured from *npc1−/−* mice were treated for 18 h with vehicle or 10 µM Y27632 at DIV3. ROCK inhibition significantly reduced growth cone collapse (48±1% vs. 80±2%; p<0.01, n = 100 growth cones) and increased p-p53 immunoreactivity in axons and growth cones in hippocampal neurons cultured from *npc1−/−* mice (Fig. 8).

**Figure 9:**
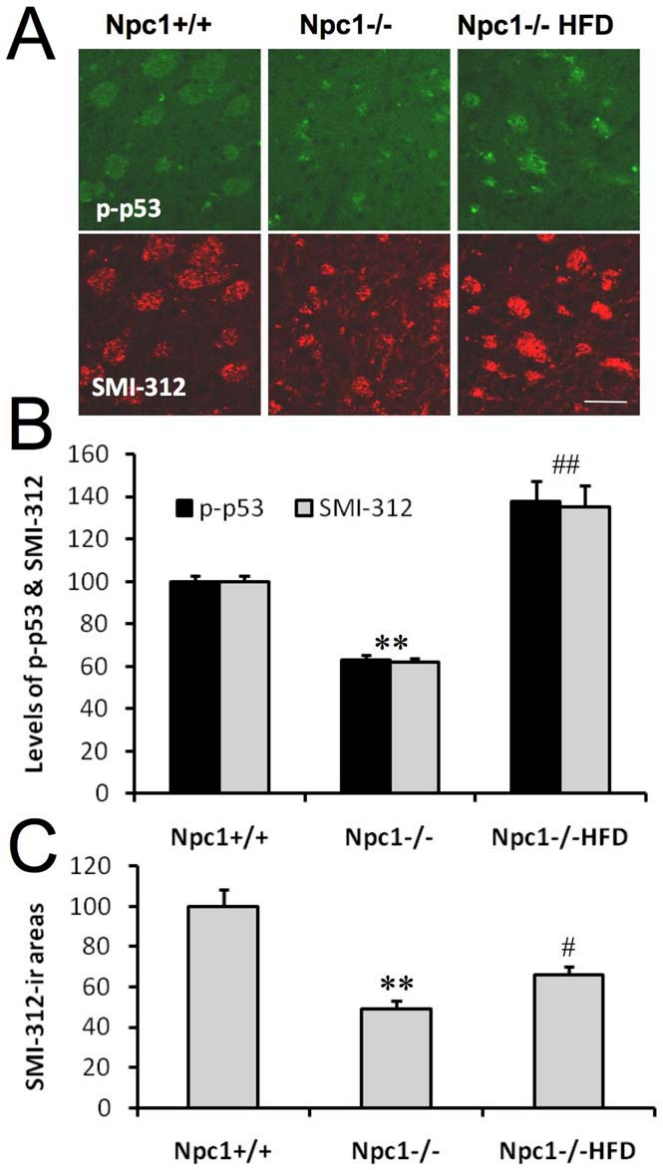
ROCK inhibition increases p-p53 and neurofilament immunoreactivity in striatal axons in developing *npc1−/−* mice. Immunostaining was performed with anti-p-p53 (green) and anti-neurofilament (SMI-312; red) antibodies in coronal brain sections from *npc1+/+* or *npc1−/−* mice treated with vehicle or hydroxyfasudil monohydrochloride (*npc1−/−*HFD). **A.** Representative images containing fasciculated bundles in the caudoputamen. **B&C.** Quantification of levels of p-p53 and SMI-312 immunoreactivity (**B**) and SMI-312 immunoreactive (SMI-312-ir) areas (**C**) in images shown in A. ** indicates p<0.01 compared to *npc1+/+* mice and # and ## indicate p<0.05 and 0.01 respectively compared to vehicle treated *npc1−/−* mice. Scale bar = 50 µm.

To further confirm that ROCK inhibition could be beneficial to axonal development in *npc1−/−* mice *in vivo*, we used another ROCK inhibitor, hydroxyfasudil monohydrochloride that has been shown to cross the blood-brain-barrier and reduce ischemia-induced brain damage [26]. Continuous administration of this ROCK inhibitor for 21 days not only increased p-p53 immunoreactivity but also increased the number of axonal neurofilaments, as revealed by staining with SMI-312 antibody, especially in corpus callosum and striatum (Fig. 9A&B). Furthermore, ROCK inhibition also significantly increased SMI-312-immunopositive areas (Fig. 9C), suggesting that this inhibitor improved axonal development.

### Inhibition of protein synthesis blocked U18666A-induced RhoA up-regulation and growth cone collapse

**Figure 10:**
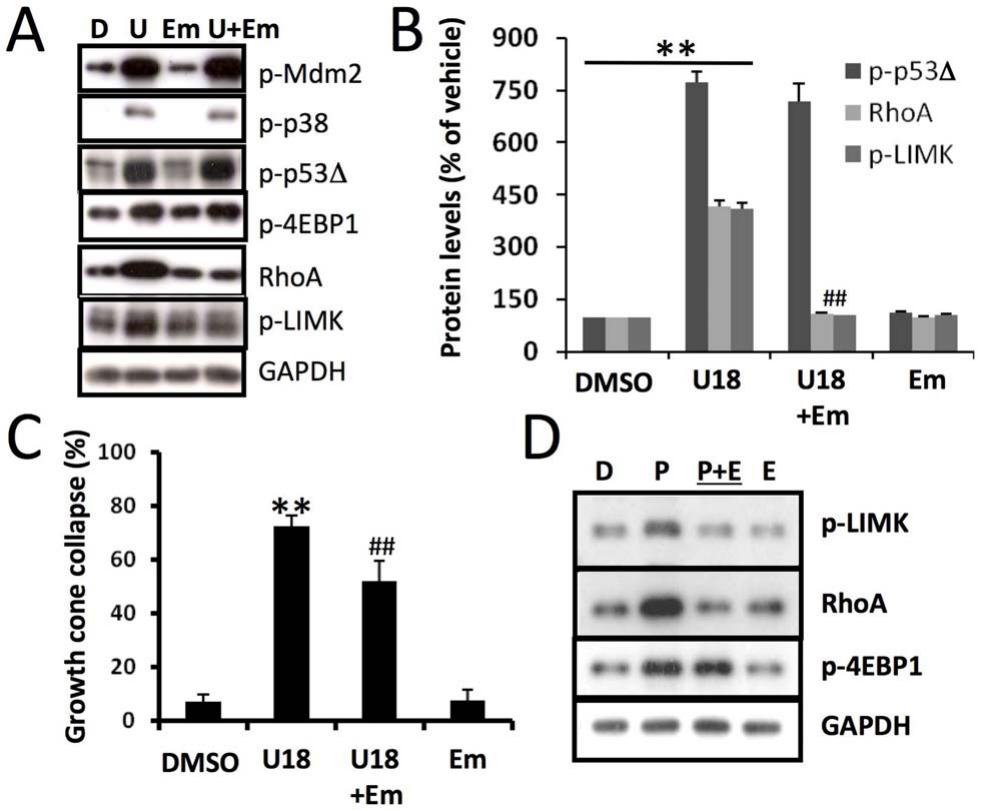
Inhibition of protein synthesis blocks U18666A-induced increase in RhoA and growth cone collapse. **A&B.** Immunoblotting analysis of various proteins in cultured cortical neurons treated with DMSO (D) or U18666A (U) in the presence or absence of the protein synthesis inhibitor ementine (Em). **A.** Representative images of immunoblots probed with antibodies against ubiquitin (Ubi), phospho-Mdm2 (p-Mdm2), phospho-p38 MAPK (p-p38), phospho-p53 (p-p53), RhoA, phospho LIM Kinase (p-LIMK), and GAPDH (loading control). **B.** Quantitative data of p-p53Δ, RhoA, and p-LIMK (**p<0.01 as compared to DMSO-treated, ##p<0.01 as compared to U18666A-treated; n = 3–6 from 3 individual experiments). **C.** Emetine application also significantly reduced U18666A treatment-induced growth cone collapse (n = 100 growth cones; ** p<0.001 as compared to DMSO-treated growth cones and ##p<0.01 as compared to U18666A-treated). **D.** Treatment with p53 inhibitor, pifithrin-μ (**P**) induced rapid increase in levels of RhoA and p-LIMK; both events were blocked by emetine (**E**) treatment.

**Figure 11:**
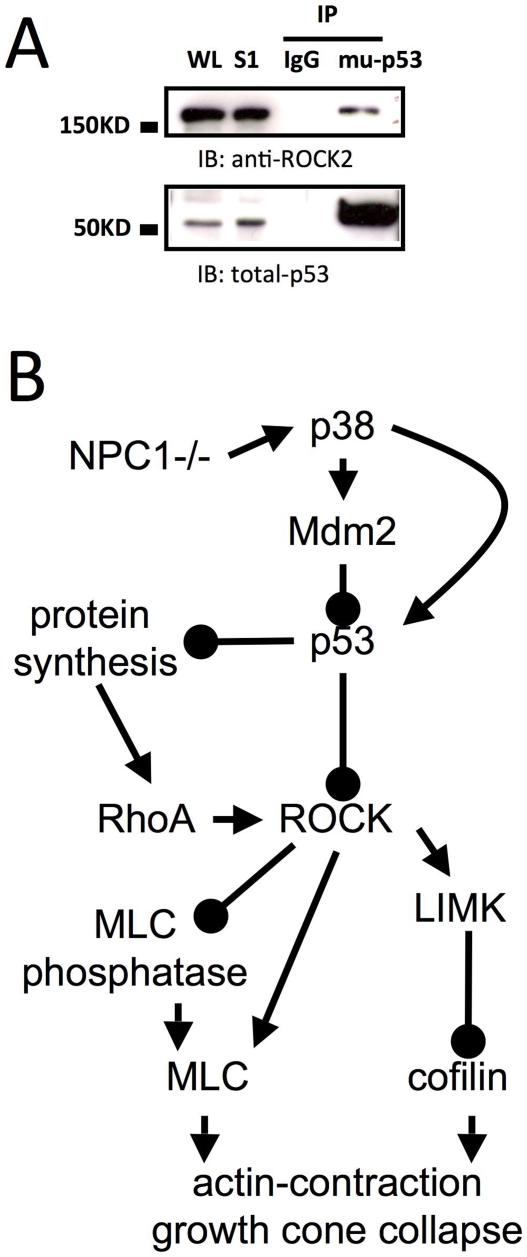
Potential signaling pathways involved in axonal pathology induced by genetic or pharmacological disruption of cholesterol homeostasis. **A.** p53 directly interacts with ROCK. Cortical neurons cultured from wild-type mice were collected on DIV4 and processed for immunoprecipitation (IP) with anti-mu-p53 antibodies (monoclonal made in mice) or control mouse IgG; immunoblots (IB) were probed with anti-p53 or anti-ROCK2 antibodies (both are rabbit polyclonal). WL, whole lysates. **B.** Perturbation of cholesterol transport, either genetically or pharmacologically, induces abnormal p38 MAPK activation, which then activates Mdm2 resulting in p53 degradation. p53 degradation disinhibits ROCK and stimulates local synthesis of RhoA leading to further increase in ROCK activation. ROCK phosphorylates and activates LIMK, leading to phosphorylation and inactivation of cofilin, which favors stabilization of filamentous actin (F-actin). On the other hand, numerous studies have shown that ROCK activation increases myosin light chain (MLC) phosphorylation through direct phosphorylation or indirectly through inhibition of MLC phosphatase-mediated dephosphorylation of MLC. Phosphorylation of MLC promotes its binding to F-actin and stimulates F-actin contraction, leading to growth cone collapse. Arrows indicate stimulation, while filled circles represent inhibition.

Emerging evidence indicates that rapid protein synthesis in axons and growth cones regulates growth cone behavior [see [27] for a recent review]. Wu et al [28] recently reported that RhoA transcripts are localized in developing axons and growth cones and that intra-axonal translation of the small GTPase is necessary and sufficient for semaphorin 3A-mediated growth cone collapse. We therefore tested whether U18666A-induced growth cone collapse was associated with increased RhoA synthesis. U18666A treatment of cultured neurons from wild-type mice rapidly increased levels of phosphorylated 4EBP1 (p-4EBP1), a widely used marker of protein synthesis initiation (Fig. 10). Immunohistochemical studies confirmed that p-4EBP1 levels were increased in axons and growth cones (Fig. S6). Pre-treatment with emetine, a protein synthesis inhibitor, significantly reduced U18666A-induced increase in RhoA levels (Fig. 10). Emetine pretreatment also significantly reduced U18666A-induced phosphorylation of LIMK and growth cone collapse, suggesting that local RhoA synthesis may contribute to ROCK-dependent growth cone collapse (Fig. 10). Emetine treatment did not affect U18666A-induced changes in levels of p-Mdm2, p-p38, and p-p53Δ (Fig. 10A), indicating that RhoA protein synthesis is a downstream event. To further test the idea that p53 could interfere with ROCK signaling by suppressing RhoA synthesis, we treated wild-type cortical neurons with the p53 inhibitor pifithrin-μ in the presence or absence of emetine pre-treatment. Immunoblotting results indicated that p53 inhibition induced a rapid increase in levels of RhoA and p-LIMK; both events were blocked by emetine pretreatment (Fig. 10D). P53 inhibition also increased levels of p-4EBP1, further supporting the notion that p53 tonically inhibits protein synthesis. Finally, immunoprecipitation experiments revealed a direct association between p53 and ROCK2, the main isoform of ROCK in brain (Fig. 11A), suggesting that p53 directly interacts with ROCK and possibly inhibits its kinase activity.

## Discussion

Although axonal pathology and hypomyelination are prominent features of both human NPC and mouse models of NPC, the mechanisms linking NPC gene mutations to axonal pathology remain to be understood. Our results showed for the first time that hippocampal and cortical neurons cultured from *npc1−/−* embryos exhibited abnormal axonal development with a high rate of growth cone collapse that was associated with a down-regulation of phosphorylated p53 in axons and growth cones. Decreased axonal levels of p-p53 were also found in developing brains of *npc1−/−* mice, indicating that these changes did not result from *in vitro* cultured conditions but were consequences of the genetic Npc1 deficiency. Both increased growth cone collapse and decreased p-p53 levels in neurons cultured from *npc1−/−* mice were significantly reduced by ROCK inhibition. Furthermore, *in vivo* ROCK inhibition not only increased axonal p-p53 levels but also increased axonal neurofilaments and the thickness of fasciculated bundles, indicating that the p-p53-ROCK pathway is critically involved in postnatal axonal development. Our results further indicated that a similar high rate of growth cone collapse was produced in cultured neurons from wild-type mice by pharmacological perturbation of cholesterol transport and was associated with degradation of p-p53 mediated by activation of p38 MAPK and Mdm2. Pharmacological perturbation of cholesterol transport also resulted in rapid increase in RhoA levels and in ROCK activation, suggesting that genetic and pharmacological disruption of cholesterol transport-induced growth cone collapse share common signaling pathways.

### P38 MAPK-mediated activation of Mdm2 is responsible for p53 degradation following perturbation of cholesterol homeostasis

Levels of p53 are regulated mainly by Mdm2, an E3 ligase, which triggers p53 ubiquitination followed by its degradation in proteasomes [29], [30], [31], [32], [33]. Subcellular localization and activity of Mdm2 are regulated by phosphorylation at various residues, including Ser166 [34], [35]. Mdm2 phosphorylation at Ser166, mediated mainly by Akt/PKB, enhances its binding to p53 and increases p53 degradation, whereas Mdm2 dephosphorylation releases and stabilizes p53 [35], [36]. Recently, it has been shown that Mdm2 is also phosphorylated by Pim kinase at Ser166 and Ser186 [37]; however, the function of this posttranslational modification is not clear. P38 MAPK phosphorylates both p53 [20] and Mdm2 [21], [22]. Previous work has shown that p38 MAPK was present in axonal growth cones of retinal neurons and that its inhibition blocked L-α-lysophosphatidic acid-induced, but not semaphorin 3A-induced, growth cone collapse [38]. The role of p38 MAPK in L-α-lysophosphatidic acid-induced growth cone collapse has been linked to increased local protein synthesis and protein degradation [38]. A recent study reported that abnormal p38 MAPK activation was responsible for Npc1 deficiency-induced impairment in self-renewal and differentiation of neural stem cells from *npc1−/−* mice [39]. Our results showed that inhibition of intracellular cholesterol transport rapidly activated p38 MAPK, suggesting that this kinase might play a critical role in NPC axonal pathology. This possibility was further supported by results obtained by inhibiting p38 MAPK with a specific inhibitor, SB203580 or with p38 specific siRNAs; both manipulations significantly reduced growth cone collapse induced by cholesterol transport inhibition. P38 MAPK suppression also reduced levels of phosphorylated/active Mdm2 and truncation of p-p53. Although p38 MAPK-mediated phosphorylation of p53 at Ser15 has been reported [40], phosphorylation of Mdm2 at Ser166 is mainly mediated by Akt/PKB [36]. We previously observed increased Akt activation in brains of *npc1−/−* mice [41]; whether p38 MAPK activates Mdm2 directly or indirectly through Akt remains to be determined. Nevertheless, our results indicate that cholesterol perturbation induces p38 MAPK activation, which in turn activates Mdm2 and leads to p53 degradation. Consequently, Mdm2 inhibition significantly reduced p53 truncation and growth cone collapse.

### P53 maintains growth cones by suppression of Rho kinase

ROCK plays critical roles in regulating cell motility and growth cone behavior. Abnormal activation of ROCK has been linked to various neurodegenerative diseases and spinal cord injury [42]. We previously showed that growth cone collapse induced by p53 inhibition was reversed by ROCK inhibitors, suggesting that p53 promotes growth cone growth by inhibiting ROCK [17]. Results from the present study showed that ROCK inhibitors significantly reduced growth cone collapse in both genetic and chemical models of NPC-type abnormal axonal development *in vitro* and *in vivo*. These results therefore not only confirm our previous conclusion that p53 promotes growth cone motility by inhibiting ROCK, but further strengthen our hypothesis that de-regulation of p53 contributes to axonal pathology in NPC. Our results indicating that U18666A treatment increased levels of phosphorylated LIM kinase, a downstream substrate of ROCK, and that the increase was blocked by Y27632 provided direct evidence that ROCK was activated under these conditions. ROCK activity is negatively regulated by its C-terminal domain, which folds back to form an auto-inhibitory loop on the kinase domain, thereby maintaining the enzyme in an inactive state [43]. Binding of RhoA to ROCK is believed to disrupt the negative auto-inhibition and to activate ROCK [42]. Since p53 over-expression significantly reduced U18666A treatment-induced growth cone collapse, it is tempting to speculate that p53, especially in its phosphorylated form, can bind to and block the structural transformation of ROCK from inactive to active, an argument that is in good agreement with our immunoprecipitation results, which indicated that p-p53 directly binds to ROCK. Emerging evidence indicates that ROCK can also be directly activated by intracellular second messengers, such as arachidonic acid [44] and sphingosylphosphorylcholine [45], independently of Rho proteins. Since both arachidonic acid and sphingosylphosphorylcholine are lipid metabolites, it is possible that they participate in the abnormal activation of ROCK in NPC through p38 MAPK activation.

### Increased local protein synthesis of RhoA contributes to cholesterol perturbation-induced growth cone collapse

U18666A treatment of wild-type cultured neurons induced a rapid increase in levels of RhoA and phosphorylated 4EBP1, and these effects were blocked by the protein synthesis inhibitor, emetine. Emetine also significantly reduced U18666A-induced growth cone collapse. Together, these results suggest that local RhoA synthesis participates in growth cone collapse produced by perturbation of cholesterol transport. These results are in agreement with the emerging notion that rapid protein synthesis in axons and growth cones regulates growth cone behavior [see [27] for a recent review]. Since emetine treatment did not affect levels of p-Mdm2, p-p38, and p-p53Δ, RhoA/ROCK-mediated growth collapse is downstream from Mdm2 and p38 MAPK. Moreover, inhibition of p38 MAPK or Mdm2 partially reduced U18666A-induced increase in RhoA and significantly suppressed ROCK activation, indicating that, in addition to direct suppression of ROCK, p53 may also inhibit the local translation of RhoA. Indeed, p53 suppression by pifithrin-μ resulted in a rapid increase in p-4EBP1 and RhoA levels, and both effects were blocked by emetine. Increased RhoA synthesis was associated with increased levels of p-LIMK, suggesting that p53 suppresses ROCK signaling by inhibiting RhoA synthesis. Corroborating this notion, over-expression of p53 resulted in rapid dephosphorylation of 4EBP1, inhibition of ribosomal protein S6 kinase and inhibition of translation initiation [46]. The signaling pathways linking lack of NPC1 proteins (or perturbations of cholesterol transport) to growth come collapse are summarized in Figure 11B.

The existing dogma in cell biology is that p53 is normally held dormant in cells and is activated when cells encounter a variety of stress signals. However, emerging evidence indicates that p53 also functions under stress-free conditions and in transcription-dependent and -independent manners. Our results for the first time demonstrate that p53 deregulation participates in abnormal axonal development in *npc1−/−* mice, and identify the signaling pathway involved in this process. As p38 MAPK and Mdm2 have also been shown to participate in pathogenesis associated with other neurodegenerative diseases, our results could have significant implications for a better understanding of a wide range of neurodegenerative diseases. Finally, our results suggest that several elements of this pathway could provide novel targets for potential drug development for the treatment of NPC and other diseases associated with axonal pathology.

## Materials and Methods

### Animals

A breeding colony of Npc1^NIH^ heterozygous mice on BALB/c background purchased from Jackson Laboratory (Bar Harbor, MA), was established at Western University of Health Sciences. The genotype was determined with PCR method, as previous described [3]. The use of animals was conducted in accordance with the National Institutes of Health Guide for the Care and Use of Laboratory Animals and animal husbandry, care and experimental protocols were approved by the Institutional Animal Care and Use Committee (IACUC) of Western University of Health Sciences.

### Neuronal cultures

Cortical and hippocampal neurons were prepared from *npc1+/+* and *npc1−/−* embryos at embryonic day 18 (E18); time-pregnant wild-type BALB/c or *npc1+/−* mice were obtained either from Charles River Laboratories (San Diego, CA) or from our breeding colony respectively. Neurons were cultured in NeuroBasal (GIBCO, Carlsbad, CA) with 10% bovine serum albumin (BSA), 2% B27, and 1% glutamine for 3–4 days before being used.

### Chemicals and antibodies

(R)-(+)-trans-N-(4-Pyridyl)-4-(1-aminoethyl)-cyclohexanecarboxamide (Y27632) and H1152 (ROCK inhibitors), trans-4-lodo, 4′-boranyl-chalcone (Mdm2-inhibitor), SB203580 (p38 inhibitor), emetine (protein synthesis inhibitor), and U18666A (cholesterol transport inhibitor) were purchased from EMD Chemicals, Inc. (Gibbstown, NJ). Control rabbit serum, anti-E6-AP, anti-ubiquitin and anti-ROCK2 antibodies were from Sigma (St. Louis, MI). Anti-RhoA and anti-p53 antibodies were from Santa Cruz Biotechnology (Santa Cruz, CA). Anti-GAPDH antibody was from Millipore (Billerica, MA). Anti-phospho-Mdm2 (Ser166), anti-phospho-p53 (Ser15), antu-phosphor-4EBP1 (Thr37/46), anti-phospho-LIMK1, 2(Thr508/505), anti-phospho-p38 MAPK (Thr180/Tyr182), anti-p38 MAPK antibodies and a p38 MAPK siRNA kit (SignalSilence®) were from Cell Signaling Technology (Danvers, MA). Mutant conformation specific p53 (Mu-p53) antibody, Alexa488 conjugated anti-rabbit and Alexa594 conjugated anti-mouse antibodies were from Invitrogen (Carlsbad, CA).

### Expression plasmids and transfection

The expression plasmids for EGFP-p53 (originally from Invitrogen) and EGFP-p53-R175H mutant plasmids were gifts from Dr. Zhiqun Tan (University of California at Irvine). Plasmid transfection was performed as previously described [17]. Briefly, neurons were incubated with DMEM (HyClone, Logan, UT) with the addition of (per ml) 1 µg plasmid DNA, 40 µl 0.25 M CaCl_2_, and 41 µl BES (pH 7.1) for 3 h. Cultured medium was then replaced with fresh medium and neurons were further cultured for 18 to 24 h before being processed for time-lapse imaging experiments or immunostaining analysis.

### Treatment

For primary cultured neurons, chemicals (U18666A and inhibitors of various enzymes) were first dissolved in 10% DMSO before being diluted in cultured medium; final DMSO concentration was lower than 0.01%. For *in vivo* treatment, hydroxyfasudil monohydrochloride (Sigma) was dissolved in double-distilled H_2_O and injected subcutaneously at 10 mg/kg, twice a day from postnatal day 7 to day 28.

### Immunofluorescent staining

Hippocampal neurons were fixed with 4% paraformaldehyde in phosphate buffer (PB; pH 7.4) for 15 min. After washing with 1x phosphate buffer saline (PBS), cells were permeabilized with 0.05% Triton X-100 in 1xPBS for 15 min, and incubated with blocking buffer (3% BSA, 0.02% Triton X-100 in 1xPBS) for 15 min before being probed with primary antibodies. The following primary antibodies were used: anti-E6AP (1∶1000), anti-phospho-p53 (1∶250), anti-phospho-4EBP1 (1∶1000), anti-phospho-Mdm2 antibody (1∶250), anti-phospho-p38 antibody (1∶250), anti-RhoA antibody (1∶1500). All primary antibodies were diluted in blocking buffer and incubated at 4°C for 18 h. After 6 washes (6×10 min) with 1xPBS at room temperature, cells were incubated with secondary antibodies, Alexa488-anti-rabbit (1∶500) or Alexa594-anti-mouse (1∶500); both antibodies were diluted in blocking buffer and incubated at room temperature for 1 h. Cells were then washed with 1xPBS (6×10 min) and sealed with mounting medium (Vectashield; Vector Laboratories, Inc., Burlingame, CA) containing 4′,6′-diamidino-2-phenylindole (DAPI) to stain nuclei. Immunofluorescent signal was detected with a Nikon confocal microscope (Nikon TE 2000U with D-Eclipse C1 system; Melville, NY).

### Filipin staining

Filipin has been demonstrated to specifically stain free cholesterol since treatment with cholesterol oxidase results in a complete loss of fluorescence [19]. After immunostaining with anti-E6-AP and anti-p-p53 antibodies and corresponding secondary antibodies conjugated with either Alexa Fluor® 594 or Alexa Fluor® 488, neurons were washed with 1xPBS and incubated in the dark with 375 µg/ml filipin in 1xPBS for 2 h at room temperature. Neuronal cultures were then washed again with 1xPBS before being examined with confocal microscopy.

### Perfusion and Immunohistochemistry

Mice were perfused with freshly prepared 4% paraformaldehyde in 1xPBS. Brains were then removed and post-fixed in 4% paraformaldehyde for 16 h followed by incubation with graded sucrose solutions. Brains were sectioned into 30 µm coronal sections with a microtome. Floating sections were processed for immunostaining as described previously [7]. Briefly, sections were incubated with rabbit anti-p-p53 (1∶250) and mouse anti-pan axonal neurofilament (SMI-312, 1∶500; Covance) antibodies in 5% horse serum diluted in 0.1M PB overnight at 4°C. After three washes, sections were incubated with Alexa Fluor® 488 conjugated goat anti-rabbit and Alexa Fluor® 594 conjugated goat anti-mouse secondary antibodies. After four more washes, sections were then mounted onto Superfrost® plus slides (VWR, West Chester, PA) and confocal images were acquired by using the Nikon microscope. Quantification of p-p53 and neurofilament immunoreactivity in fasciculated bundles in the striatum was performed by using NIH ImageJ software. Briefly, images of the caudoputamen from different animals were taken at the same coronal level using the same acquisition parameters. Analyzed area consisted of 450 µm x420 µm that was taken from two sections per mouse; three different mice were used for each experimental group. Means of integrated density and areas were quantified and expressed as percentage of values from *npc1+/+* mice.

### Quantification of growth cone morphology and immunoreactivity

Confocal images were taken using the 60x oil-immersion objective. About 20–30 images were randomly selected from each culture dish (20 mm in diameter); at least 4–6 dishes from 3–6 independent culture preparations/experiments were used for each experimental group. Within an experiment, cultures used for different experimental groups and designed for comparison were stained simultaneously and imaged with the same acquisition parameters. Quantification was done blindly by multiple researchers. Growth cones with less than 1 filopodium were considered collapsed; 100 growth cones were quantified for each experimental group. Image J software was used to quantify immunoreactivity intensity of p-p53 and RhoA in axons and growth cones; the “total integrated density” was used instead of “average intensity”. Briefly, individual growth cones were outlined manually and the total integrated density was measured using Image J software. For quantification of immunoreactivity in axons, a 50 µm fragment of axons from the neck of growth cones towards the cell body was selected and integrated density measured.

### Immunoprecipitation and immunoblotting procedures

For immunoprecipitation, cultured cortical neurons were lysed in lysis buffer [0.05 M Tris base, 0.9% NaCl, pH 7.6, and 0.5% Triton X-100 plus Protease Inhibitors Cocktail (1∶100; EMD Biosciences) and phosphatase inhibitor cocktails (1∶500; Sigma)]. Lysates were centrifuged at 16,000×*g* for 30 min at 4°C. Supernatant were then cleared with a mixture of protein A/G-agarose beads (each 50%) for 1 h at 4°C, and after a brief spin, pellets were discarded. A small portion of the supernatants was used as input. The reminder of the supernatant was immunoprecipitated overnight with control IgG or Tau1 antibodies. Immunoprecipitates were captured by incubation with protein A/G-agarose beads for 3 h at 4°C. After several washes, the beads were resuspended in 2xSDS sample buffer [4% sodium dodecyl sulfate (SDS), 100 mM Tris-HCl (pH 6.8), 10% β-mercaptoethanol, 20% glycerol and 0.2% bromophenol blue] and boiled for 10 min. The resulting proteins were separated by SDS-PAGE, and transferred to polyvinylidene difluoride membranes for immunoblotting using previously described protocols [17].

### Statistics

All experiments were performed at least 3 times with independent culture preparations. Results were expressed as means ± SEM, and p values were determined by one-way ANOVA followed by post-hoc analysis; p values less than 0.05 were considered statistically significant.

## Supporting Information

Figure S1 Decreased axonal p-p53 immunoreactivity in the striatum of Npc1−/− mice. Immunofluorescent staining with anti-p-p53 (green) and anti-axon specific neurofilament (SIM-312; red) was performed on coronal brain sections from 2 week-old Npc1+/+ and Npc1−/− mice. In the striatum, p-p53 immunoreactivity was clearly reduced in axonal bundles containing axonal neurofilaments in Npc1−/− mice as compared to wild-types. p-p53 immunoreactivity was also present in oligodendrocytes. Scale bar = 50 µm.
(6.53 MB TIF)

Figure S2 Over-expression of wild-type p53 blocks U18666A-induced growth cone collapse. DIV3 hippocampal neurons from wild-type mice were first transfected with EGFP-vector (A), EGFP-wild-type-p53 (p53-wt; B), or EGFP-mutant-p53 (p53-mu; C); 18 h later they were treated with 5 µM U18666A for 2 min before being processed for immunostaining with anti-E6AP antibodies (red). Scale bar = 20 µm.
(4.42 MB TIF)

Figure S3 Localization of p38 MAPK and Mdm2 in axons and growth cones. DIV4 hippocampal neurons from wild-type mice were treated with DMSO or 5 µM U18666A for 2 min before being processed for immunofluorescence analysis of phosphorylated p38 (p-p38, green) and Mdm2 (p-Mdm2, green) distribution in axons and growth cones. Neurons were doubled immunostained with anti-E6AP antibodies (red). Inserts show enlarged images of growth cones. Scale bar = 50 µm.
(5.40 MB TIF)

Figure S4 ROCK inhibition with H1152 blocks U18666A-induced p-p53 decrease and rescues growth cones in cultured hippocampal neurons. Hippocampal neurons were treated on DIV4 with the ROCK inhibitor, H1152 (100 nM) for 3 h before being exposed to U18666A (U18, 5 µM) or DMSO for 2 min. Neurons were then subjected to immunofluorescence analysis of p-p53 (green) and E6-AP (red) distribution in axons and growth cones. Scale bar = 20 µm.
(6.77 MB TIF)

Figure S5 ROCK inhibition blocks U18666A treatment-induced decreases in “conformational mutant” p53 in axons and growth cones. DIV4 hippocampal neurons from wild-type mice were treated with DMSO or 5 µM U18666A for 2 min with or without pre-incubation with 10 µM Y27632. Neurons were then immunostained with anti-p-p53 (green) antibodies and a “conformational mutant” p53 specific antibody (mu-p53, red). Scale bar = 20 µm.
(3.21 MB TIF)

Figure S6 Localization of phospho-4EBP1 in axons and growth cones. DIV4 hippocampal neurons from wild-type mice were treated with DMSO or 5 µM U18666A for 2 min before being processed for immunofluorescence analysis of phosphorylated 4EBP1 (p-4EBP1, green) and E6AP (red) distribution in axons and growth cones. Inserts show enlarged images of growth cones. Scale bar = 50 µm.
(3.34 MB TIF)
