# Supplementary figures and images for: Cholesterol Perturbation in Mice Results in p53 Degradation and Axonal Pathology through p38 MAPK and Mdm2 Activation
Source: PLoS One. 2010 Apr 6;5(4):e9999. doi: 10.1371/journal.pone.0009999 (PMC2850309; doi:10.1371/journal.pone.0009999)

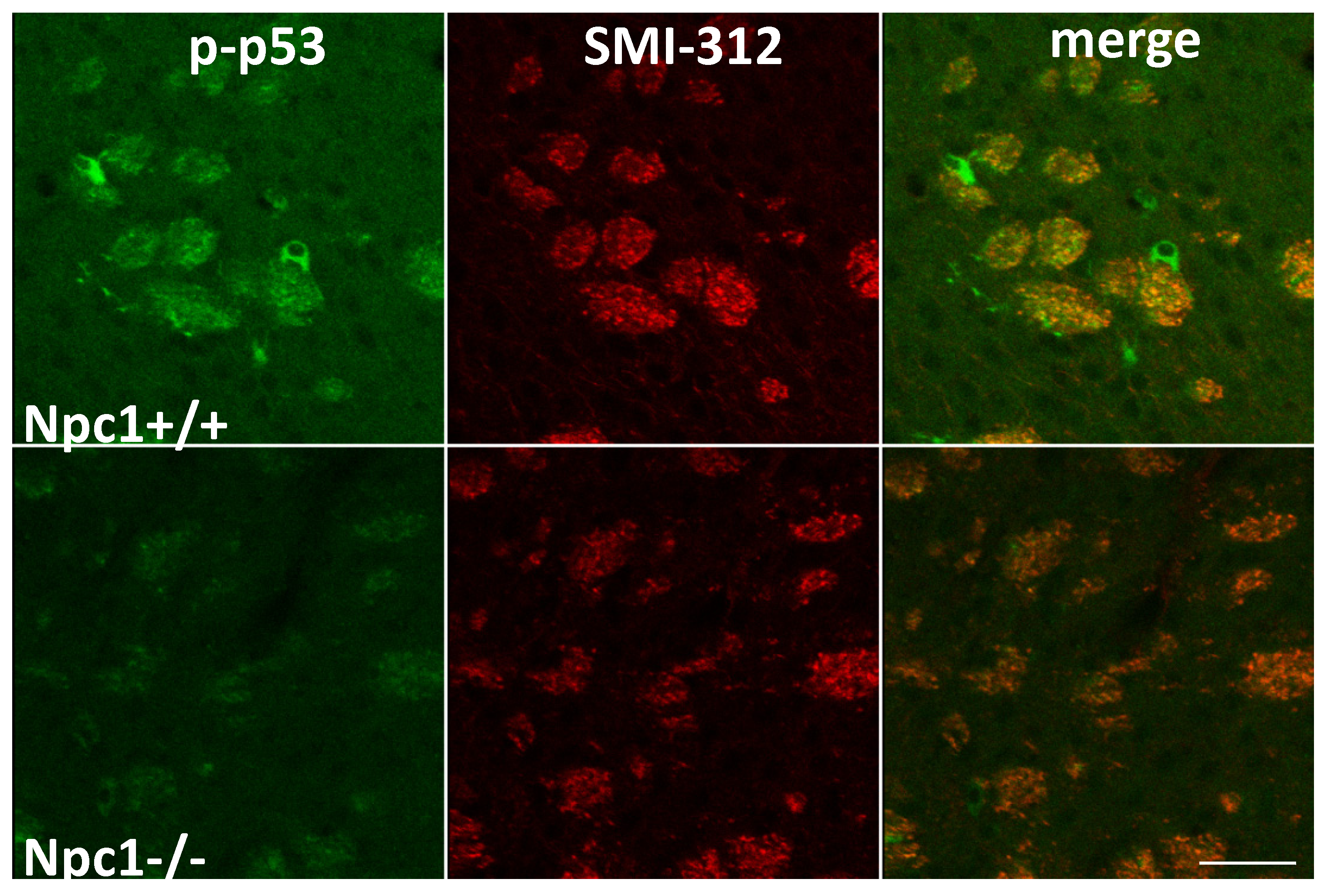

Supplement: Figure S1 — Decreased axonal p-p53 immunoreactivity in the striatum of Npc1−/− mice. Immunofluorescent staining with anti-p-p53 (green) and anti-axon specific neurofilament (SIM-312; red) was performed on coronal brain sections from 2 week-old Npc1+/+ and Npc1−/− mice. In the striatum, p-p53 immunoreactivity was clearly reduced in axonal bundles containing axonal neurofilaments in Npc1−/− mice as compared to wild-types. p-p53 immunoreactivity was also present in oligodendrocytes. Scale bar = 50 µm. (6.53 MB TIF) [file pone.0009999.s001.tif]

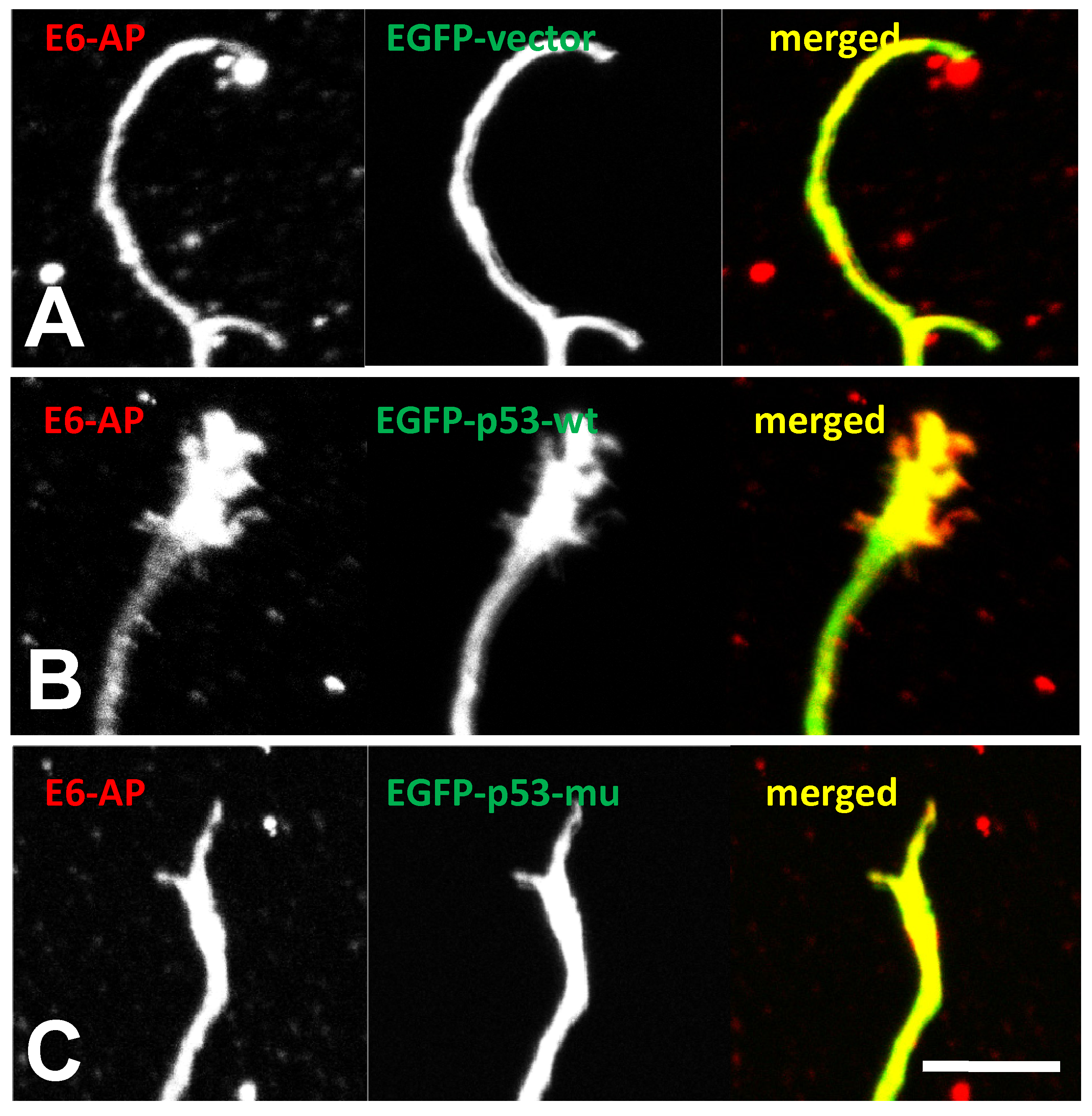

Supplement: Figure S2 — Over-expression of wild-type p53 blocks U18666A-induced growth cone collapse. DIV3 hippocampal neurons from wild-type mice were first transfected with EGFP-vector (A), EGFP-wild-type-p53 (p53-wt; B), or EGFP-mutant-p53 (p53-mu; C); 18 h later they were treated with 5 µM U18666A for 2 min before being processed for immunostaining with anti-E6AP antibodies (red). Scale bar = 20 µm. (4.42 MB TIF) [file pone.0009999.s002.tif]

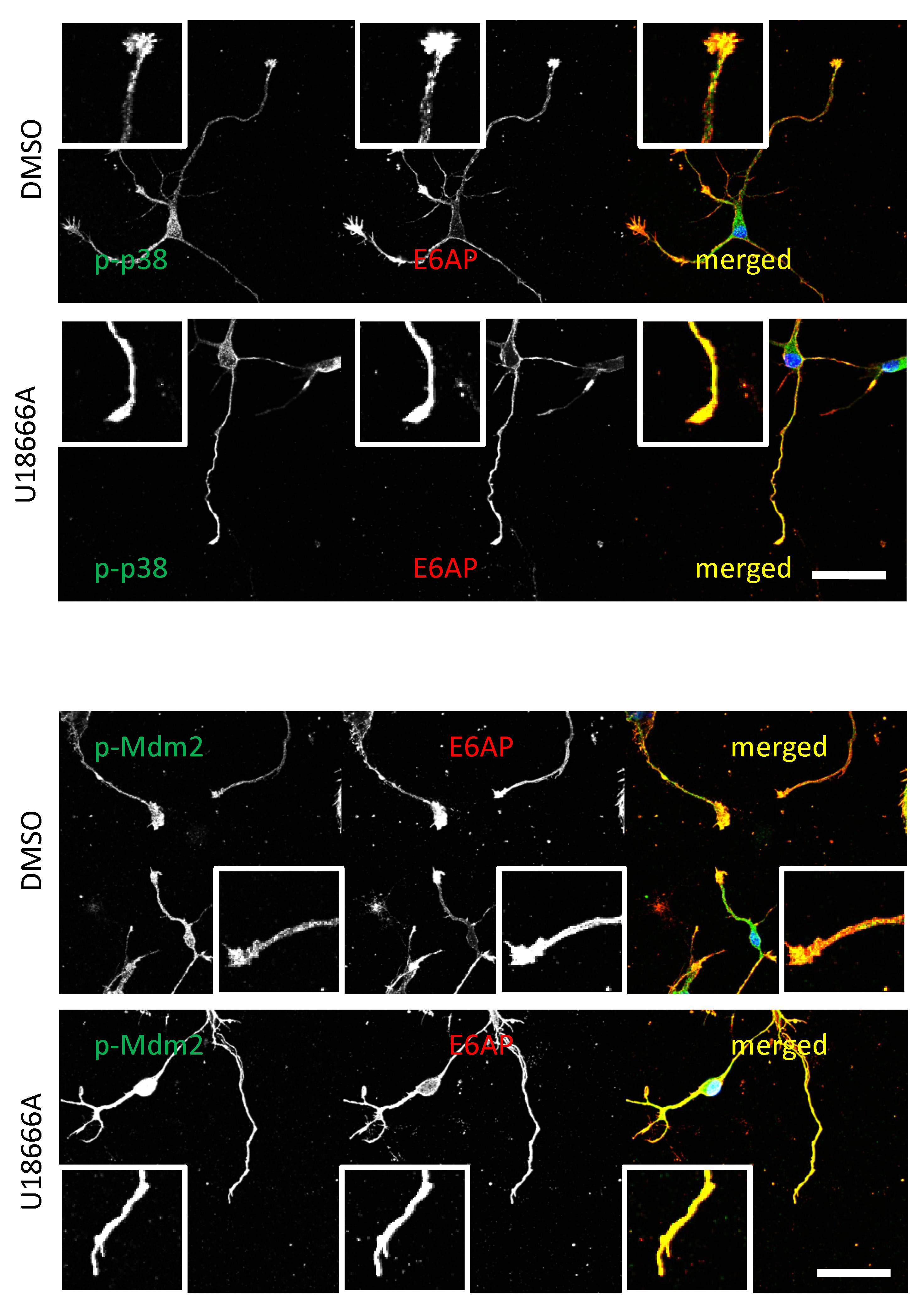

Supplement: Figure S3 — Localization of p38 MAPK and Mdm2 in axons and growth cones. DIV4 hippocampal neurons from wild-type mice were treated with DMSO or 5 µM U18666A for 2 min before being processed for immunofluorescence analysis of phosphorylated p38 (p-p38, green) and Mdm2 (p-Mdm2, green) distribution in axons and growth cones. Neurons were doubled immunostained with anti-E6AP antibodies (red). Inserts show enlarged images of growth cones. Scale bar = 50 µm. (5.40 MB TIF) [file pone.0009999.s003.tif]

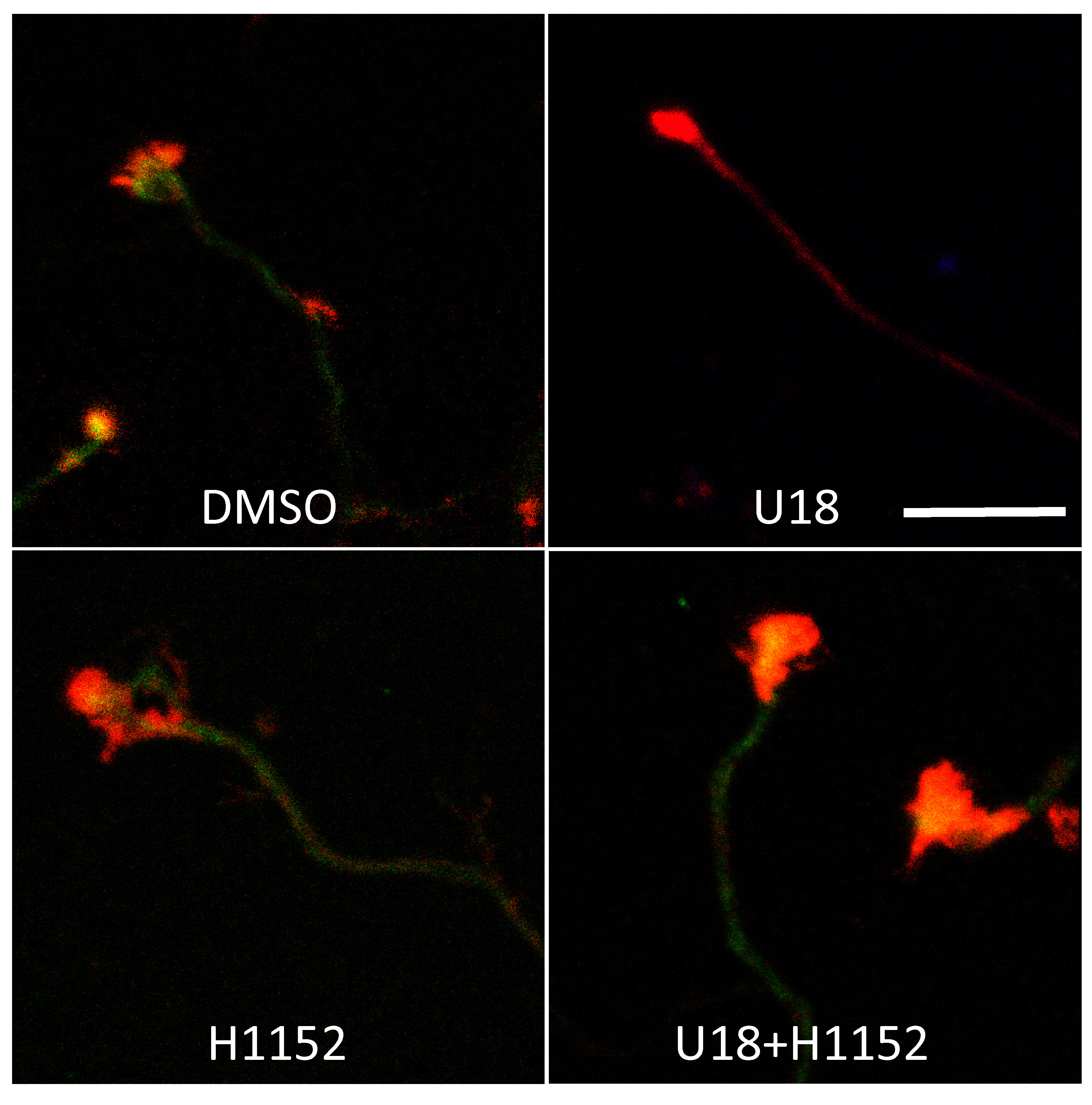

Supplement: Figure S4 — ROCK inhibition with H1152 blocks U18666A-induced p-p53 decrease and rescues growth cones in cultured hippocampal neurons. Hippocampal neurons were treated on DIV4 with the ROCK inhibitor, H1152 (100 nM) for 3 h before being exposed to U18666A (U18, 5 µM) or DMSO for 2 min. Neurons were then subjected to immunofluorescence analysis of p-p53 (green) and E6-AP (red) distribution in axons and growth cones. Scale bar = 20 µm. (6.77 MB TIF) [file pone.0009999.s004.tif]

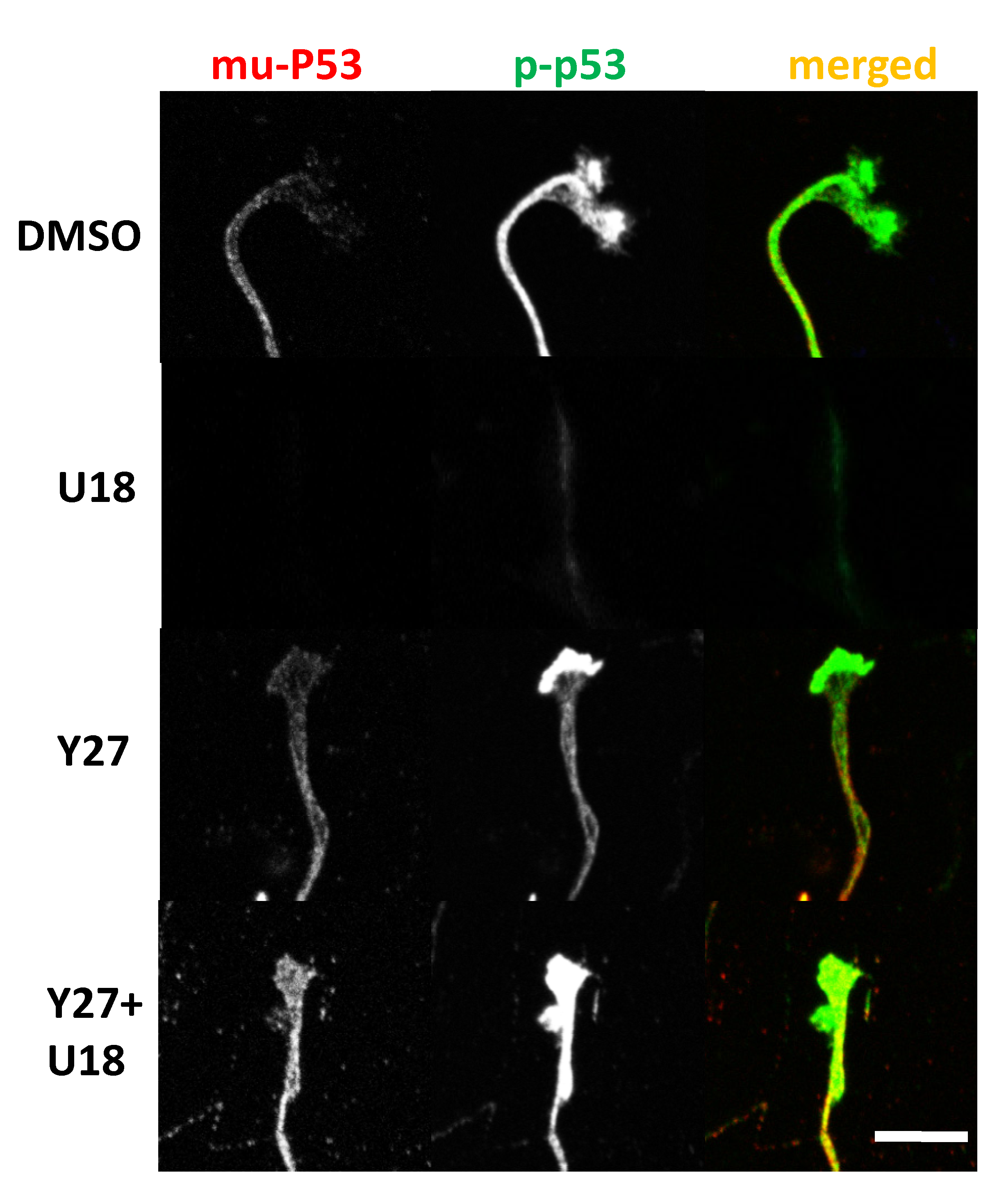

Supplement: Figure S5 — ROCK inhibition blocks U18666A treatment-induced decreases in “conformational mutant” p53 in axons and growth cones. DIV4 hippocampal neurons from wild-type mice were treated with DMSO or 5 µM U18666A for 2 min with or without pre-incubation with 10 µM Y27632. Neurons were then immunostained with anti-p-p53 (green) antibodies and a “conformational mutant” p53 specific antibody (mu-p53, red). Scale bar = 20 µm. (3.21 MB TIF) [file pone.0009999.s005.tif]

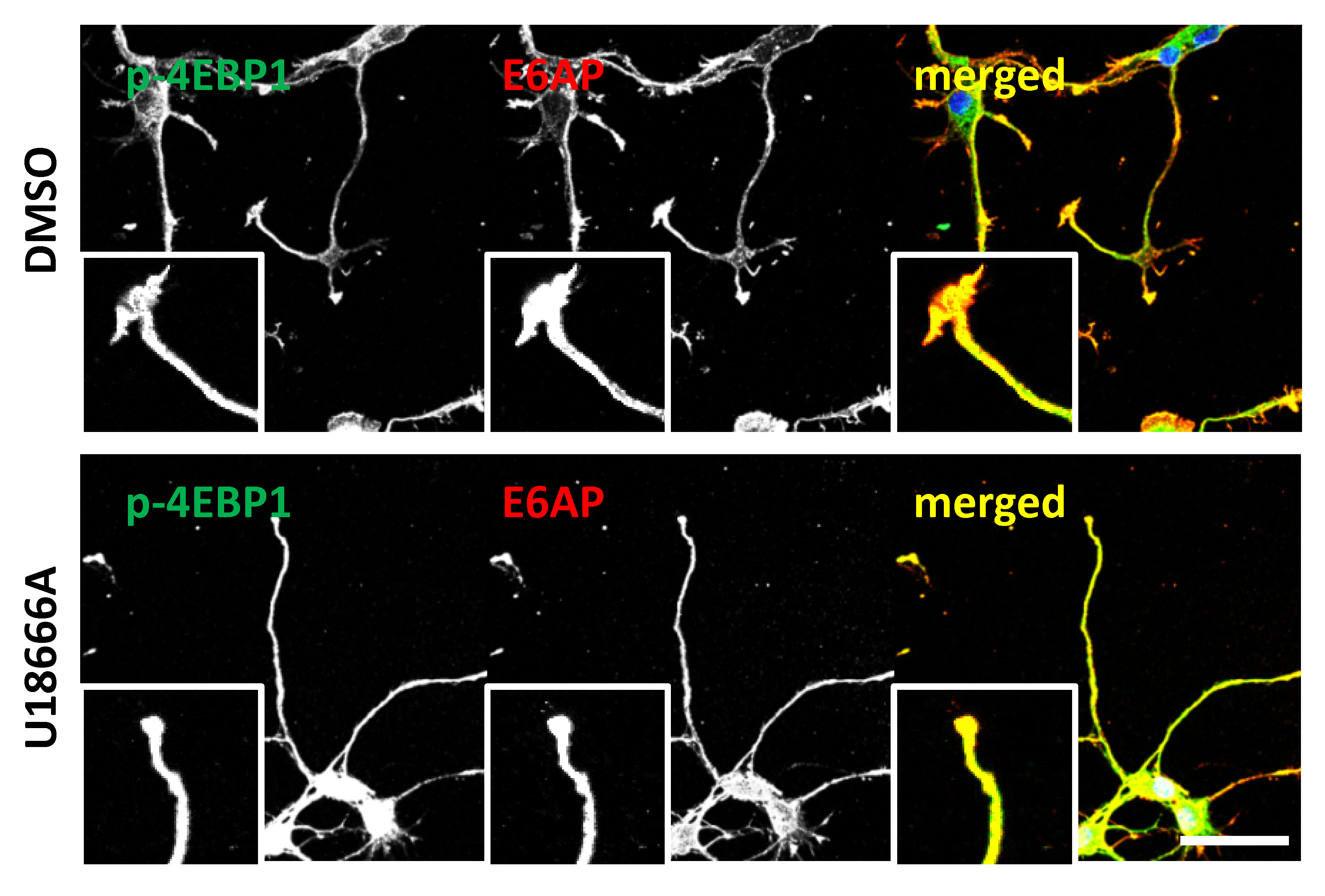

Supplement: Figure S6 — Localization of phospho-4EBP1 in axons and growth cones. DIV4 hippocampal neurons from wild-type mice were treated with DMSO or 5 µM U18666A for 2 min before being processed for immunofluorescence analysis of phosphorylated 4EBP1 (p-4EBP1, green) and E6AP (red) distribution in axons and growth cones. Inserts show enlarged images of growth cones. Scale bar = 50 µm. (3.34 MB TIF) [file pone.0009999.s006.tif]
